# All-trans-retinoic acid (ATRA) plus oxaliplatin plus 5-fluorouracil/leucovorin (FOLFOX) versus FOLFOX alone as palliative chemotherapy in patients with advanced hepatocellular carcinoma and extrahepatic metastasis: study protocol for a randomized controlled trial

**DOI:** 10.1186/s13063-019-3349-9

**Published:** 2019-04-29

**Authors:** Jie Shi, Juxian Sun, Chang Liu, Zongtao Chai, Nanya Wang, Hui Zhang, Shuqun Cheng

**Affiliations:** 1 Department of Hepatic Surgery VI, Eastern Hepatobiliary Surgery Hospital, Second Military Medical University, Changhai Rd No.225, Shanghai, 200438 China; 2 Department of Cancer Center, First Hospital of Jilin University, Xinmin Rd No71, Changchun, 130021 Jilin Province China; 3 Department of Surgery of Hepato-Biliary & Pancreatic Tumor, Fujian Provincial Cancer Hospital, Fuma Rd No.420, Fuzhou, 350011 Fujian Province China

**Keywords:** Extrahepatic metastasis, FOLFOX4, ATRA, Palliative chemotherapy

## Abstract

**Background:**

Among patients with hepatocellular carcinoma (HCC), 85% of patients have an advanced disease stage at diagnosis and curative therapies cannot be performed. Prognosis has been quite poor as until recently there was no proven effective chemotherapy. Our group found that all-trans-retinoic acid (ATRA) could improve the efficacy of platinum in HCC in vivo and in vitro, thus we wish to validate the efficiency of ATRA in clinical practice.

**Methods:**

This is a double-blinded, 1:1 randomized, controlled, multicenter clinical trial. Three hundred and sixty-eight patients with HCC and extrahepatic metastases will receive palliative chemotherapy at the Eastern Hepatobiliary Surgery Hospital, First Hospital of Jilin University and Fujian Provincial Cancer Hospital. Subjects will be randomly assigned to one of the two arms, either ATRA + oxaliplatin + 5-fluorouracil/leucovorin (FOLFOX4) or FOLFOX4 alone. ATRA 20 mg will be given orally three times/day for 3 days prior to the initiation of FOLFOX4. ATRA will be discontinued at the end of FOLFOX4.

**Discussion:**

Overall survival rate is the primary endpoint. Secondary endpoints are time to progression according to the modified response evaluation criteria in solid tumors (mRECIST) criteria, acute and chronic adverse events, and quality of life.

**Trial registration:**

Chinese Clinical Trial Registry, ChiCTR-IIR-17012916. Registered on 9 October 2017.

**Electronic supplementary material:**

The online version of this article (10.1186/s13063-019-3349-9) contains supplementary material, which is available to authorized users.

## Background

Hepatocellular carcinoma (HCC) is the fifth most common cancer in men, the eighth in women, and the third most frequent cause of cancer-related death worldwide, with more than 626,000 new cases per year [1]. The geographic distribution is not uniform across the world: approximately 80% of cases arise in Asia and Africa where the incidence of HCC is 30–120/100,000 in men and 9–30/100,000 in women. The incidence in Europe and North America States is < 5/100,000 in men and < 3/100,000 in women and has increased in the last three decades as a result of the high prevalence of hepatitis C [2–4]. HCC commonly develops in chronic liver cell injury, which leads to inflammation, hepatocyte regeneration, liver matrix remodeling, fibrosis, and ultimately, cirrhosis [5, 6]. The major etiology of liver cirrhosis includes chronic hepatitis B virus (HBV) and hepatitis C virus (HCV) infection, alcohol consumption, steatosis, aflatoxin exposure, and diabetes mellitus, etc. In Asia, HCC is mainly due to chronic HBV infection [5]. Overall, HCC is associated with cirrhosis in about 80% of cases and is currently the leading cause of death among patients with cirrhosis [6].

In order to determine whether locally advanced, metastatic or recurrent HCCs are candidates for systemic treatment, many anti-cancer agents or combinations of agents have been tested in HCC in the last 30 years, including systemic chemotherapy, immunotherapy, and hormonal therapy [7–9]. The clinical application of more aggressive systemic chemotherapy regimens is severely limited by liver cirrhosis and compromised liver function in patients with advanced stage HCC. Systematic reviews and meta-analyses consistently demonstrate that systemic chemotherapy does not prolong survival in patients with advanced HCC. The median overall survival (mOS) ranges from 3 to 7 months in most clinical studies, regardless of different etiology, ethnic group, and standard of care across the regions [7, 10, 11].

Two recent randomized phase III studies in HCC further confirmed the limited effectiveness of chemotherapy in advanced HCC. A study conducted in Hong Kong testing doxorubicin versus cisplatin, interferon α-2b, fluorouracil, and doxorubicin (PIAF) combination showed no significant survival benefit (mOS 8.6 vs 6.83 months, not statistically significant) [12]. Another international study of nolatrexed versus doxorubicin showed lesser survival benefit of nolatrexed compared to doxorubicin (mOS 5.2 vs 7.53 months, *p* < .05) [13].

Qin et al. [14] conducted a multicenter, open-label, randomized, phase III study in mainland China, Taiwan, Korea, and Thailand, which involved 371 patients age 18–75 years, who had locally advanced or metastatic HCC and were ineligible for curative resection or local treatment. They were randomly assigned at a ratio of one to one to receive either oxaliplatin + 5-fluorouracil/leucovorin (FOLFOX4) (*n* = 184) or doxorubicin (*n* = 187). Although the study did not meet its primary endpoint, the trend toward improved overall survival (OS) with FOLFOX and the increased progression-free survival (PFS) and response rate (RR) suggests that this regimen may confer some benefit in Asian patients, but an OS benefit cannot be concluded from these data [14].

All-trans-retinoic acid (ATRA), a group of structural and functional analogues of vitamin A, exerts fundamental effects on the regulation of epithelial cell growth, differentiation, and development [15]. ATRA exerts a biological function primarily by regulating gene expression through two distinct nuclear receptors, retinoic acid receptors and retinoid X receptors, which are both composed of three subtypes (α, β, and γ). The cellular activity of ATRA is largely well-characterized and translates to the regulation of processes such as differentiation and cell death, which are critical in the outcome of malignant transformation of tissues. It is well-known that ATRA is one of the strongest and the most thoroughly studied differentiation inducers that may induce differentiation of several types of tumor cells, including stem cells. In fact, retinoid-based differentiation therapy in acute promyelocytic leukemia is one of the first successful examples of molecularly targeted treatment strategies. Our previous studies showed that ATRA can induce differentiation of HCC tumor-initiating cells (TICs) via the tumor sclerosis compound 2 (TSC2)/protein kinase B (AKT) pathway and combined treatment with ATRA and that cisplatin improves the therapeutic effect, due to elimination of TICs via ATRA-induced differentiation, in vivo and in vitro [16, 17]. Thus, ATRA may be of benefit both in cancer prevention and cancer treatment when used in combination with classic chemotherapeutic agents [18]. So far, there is no report on the efficacy and safety of ATRA and FOLFOX in the treatment of extrahepatic metastatic HCC. Therefore, our hypothesis is that administration of ATRA with FOLFOX will significantly prolong survival compared to FOLFOX alone, when used as palliative chemotherapy in patients with advanced HCC and extrahepatic metastasis.

## Study objectives

The objectives are:

1. To determine whether ATRA + FOLFOX improves overall survival, progress-free survival, time to progress, symptomatic progress, and health-related quality of life.
2. To determine the safety of ATRA given in combination with FOLFOX.

## Study design

This is a multicenter, double-blinded, placebo-controlled, randomized clinical trial in patients with advanced HCC and extrahepatic metastasis. Subjects will be randomly assigned to one of the two arms, either ATRA + FOLFOX or FOLFOX alone. Our study has been registered at http://www.chictr.org.cn (ID: ChiCTR-IIR-17012916).

**Fig. 1:**
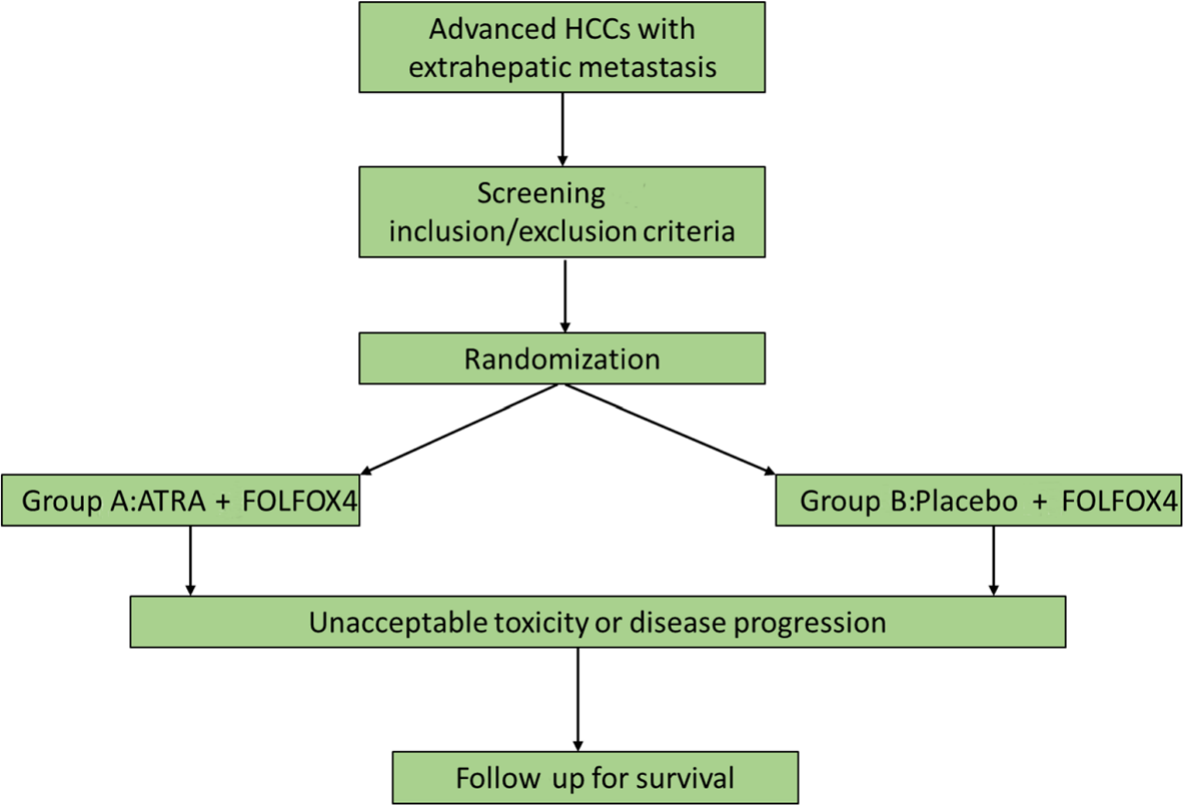
Study design. HCC, hepatocellular carcinoma

After screening for eligibility and provision of signed, informed consent, eligible subjects will be randomized in a 1:1 ratio to receive ATRA + FOLFOX4 (group A) or placebo + FOLFOX4 (group B). Randomization will be stratified by site and researchers will remain blinded to treatment assignments throughout the study (Fig. 1). The Standard Protocol Items: Recommendations for Interventional Trials (SPIRIT) checklist was utilized as a guideline for this study (Additional file 1).

The therapeutic agents to be used in this study are:

- All-trans-retinoic acid (ATRA)
- Oxaliplatin (OXA)
- 5-Fluorouracil (5FU)
- Leucovorin (LV)

The mode of administration will be as follows:

1. ATRA + FOLFOX4 (group A):
  ATRA 20 mg given orally three times/day for 3 days prior to the initiation of chemotherapy (FOLFOX4). ATRA will be discontinued at the end of FOLFOX4.
    OXA 85 mg/m^2^ given intravenously (iv), day1
    LV 200 mg/m^2^ given iv, day1 and day 2
    5FU 400 mg/m^2^ iv bolus, then 600 mg/m^2^ given iv over 22 h, day 1 and day 2.
    Repeated every 2 weeks.
2. Placebo + FOLFOX4 (group B):
  The placebo (replacing ATRA) will be given orally three times/day for 3 days prior to the chemotherapy. The placebo will then be discontinued at the end of FOLFOX4.
    OXA 85 mg/m^2^ given iv, day1
    LV 200 mg/m^2^ given iv, day1 and day 2
    5FU 400 mg/m^2^ iv bolus, then 600 mg/m^2^ given iv over 22 h, day 1 and day 2.
    Repeated every 2 weeks.

Tumor response will be evaluated in each subject every 4 weeks. Tumors will be evaluated by computed tomography (CT) or magnetic resonance imaging (MRI). Progression will be determined based on the modified response evaluation criteria in solid tumors (mRECIST) for HCC. Drug therapy will be continued until unacceptable toxicity or disease progression occurs. If the investigator (blinded to the assigned study drug) determines the subject is benefiting from the drug and the subject (blinded to the assigned study drug) is willing to continue taking the drug, the treatment may be continued beyond radiographic progression. Choice of subsequent therapies in the case of progressive disease will be at the discretion of the investigator, which should be beneficial to subjects overall. Data on subsequent treatments for HCC will be collected in the follow-up period. Requests for un-blinding will only be granted for emergency medical management. Subjects who have discontinued study therapy due to study drug toxicity or for any reason other than confirmed tumor progression will continue to have tumor assessments every 4 weeks until documented radiographic tumor progression. All randomized subjects will be followed for overall survival until the required number of events has been reached.

Treatment using the investigational agents will be discontinued due to any of the following reasons:

1. Withdrawal of informed consent (subject’s decision to withdraw for any reason).
2. Any clinical adverse event (AE), laboratory abnormality, or intercurrent illness which, in the opinion of the investigator, indicates that continued participation in the study is not in the best interest of the subject.
3. Pregnancy.
4. Loss of ability to freely provide consent due to either a psychiatric or physical (e.g. infectious disease) illness.
5. Disease progression
  Note that if the investigator (blinded to the assigned study drug) determines the subject (blinded to the assigned study drug) is benefiting from the drug (e.g. the subject may be experiencing symptomatic improvement at the time of documented radiographic progression) and the subject is willing to continue the drug, the treatment may be continued beyond radiographic progression.

## Standard best supportive care

All subjects enrolled in this clinical study should always receive standard best supportive care (SBSC), which is defined by the institutional standards of the participating clinical centers. In general, SBSC means that multi-professional attention to the patient’s overall physical, psychosocial, spiritual, and cultural needs must be available at all stages of the illness. Attention to the physical wellbeing of the subjects with HCC who are enrolled in this trial, SBSC may include but may not be restricted to: (1) blood transfusion for anemia; (2) antibiotics to control infection; (3) analgesics, including non-steroidal anti-inflammatory drugs, opioids, and corticosteroids; (4) antiemetic drugs; (5) vitamin and nutritional support; and (6) alternative medicine (including herbal medicine), except for medicine labeled as potentially having “anticancer activity” or “immunologic activity”. Localized radiation therapy to alleviate symptoms such as pain due to non-target lesions is allowed, according to institutional standards. Other palliative care will be also provided in the best interest of subjects receiving a study drug.

## Study population

The study population will comprise patients with HCC and extrahepatic metastasis that is pathologically confirmed at the Eastern Hepatobilliary Surgery Hospital, First Hospital of Jilin University and Fujian Provincial Cancer Hospital.

### Inclusion criteria

The inclusion criteria are as follows:

1. Men and women above the age of 18 years, with documented advanced HCC
2. Child-Pugh Class A or B.
3. Confirmed extrahepatic metastatic lesions located in bone, lung, brain, or abdominal lymph nodes.
4. Eastern Cooperative Oncology Group (ECOG) performance status grade 1 or lower.
5. Liver and renal laboratory tests within the following ranges:
  - Total bilirubin ≤ 1.5 times the upper normal limit (UNL) defined by each clinical laboratory at each site.
  - Glutamic-pyruvic transaminase (ALT) and glutamic oxaloacetic transaminase (AST) < 2.5 times the UNL defined by each clinical laboratory at each site.
  - Serum creatinine ≤ 1 of the UNL defined by each clinical laboratory at each site.
  - Endogenous creatinine clearance rate > 50 ml/min (calculated using the Cockcroft-Gault formulation).
6. Other laboratory tests:
  - Hemoglobin > = 90 g/L
  - Platelet count > = 100 × 10^9^/L
  - White blood cell (WBC) count > = 4.0 × 10^9^/L
7. Good cardiac function, no myocardial infarction within 6 months; well-controlled hypertension and coronary heart disease if present.

### Exclusion criteria

The exclusion criteria are as follows:

1. No extrahepatic metastatic lesion identified.
2. Hepatic encephalopathy.
3. Pregnancy or breast-feeding.
4. Hypertension that is uncontrolled even with medication.
5. Hemorrhagic disorders, active bleeding, or sepsis
6. History of HIV infection or AIDS.
7. Active or uncontrolled severe infections.
8. Congestive heart failure (CHF) New York Heart Association (NYHA) class > 2, active coronary atherothrombotic disease (CAD) or cardiac ischemia, or cardiac arrhythmias that are uncontrolled even with anti-arrhythmic therapy.
9. Inability to take oral medication.
10. Low compliance as judged by the investigators.
11. Seizure disorder requiring medication.
12. Radiotherapy, transhepatic arterial chemotherapy and embolization (TACE), or hormone therapy within 4 weeks.
13. History of drug abuse, or mental disorders that could compromise compliance.
14. Any other condition that may jeopardize patient safety and compliance.

## Informed consent

The investigator must ensure that written informed consent to participate in the study is obtained before including any individual as a subject in the study, and before conducting any study-related assessments. The investigator must provide the prospective subject, or the prospective subject’s legally authorized representative, with sufficient opportunity to consider whether to participate, and minimize the possibility of coercion or undue influence. The process is designed to (1) give the subject all the information that he/she needs, (2) ensure that the subject understands the information, and (3) give the subject a chance to consider study participation. The process should permit the subject to ask questions and exchange information freely.

Specifically, the investigator must explain to each subject all elements of informed consent as specified by China Food and Drug Administration (CFDA). This also includes explaining that photographs will be taken and may be used in publications in ways that do not identify the subject. After the explanation, the subject or legally authorized representative will voluntarily sign and date the consent/assent form if they wish to participate in the study. A copy of the consent/assent form must be provided to the subject or the subject’s legally authorized representative. A signed and dated copy of the consent/assent form must always be maintained in the investigator site file. The informed consent process must be followed, and the subject’s participation in the study, must be documented in the subject’s medical record/chart.

## Endpoints and assessment

### Primary endpoint

The primary endpoint is overall survival (OS): this is defined as the time from randomization to the time of death from any cause. Subjects who are alive at the time of the final analysis or who have become lost to follow up will be censored at the last known date that they were alive.

### Secondary endpoints

The secondary endpoints are time to progression (TTP), the objective response rate (ORR), the disease control rate (DCR), duration of response (DR), time to response (TR), and quality of life (QoL).

TTP is defined as the time (in months) from randomization to the time of radiographic disease progression. Subjects without progression will be censored at their last tumor assessment date. Subjects who have no on-study tumor assessments will be censored at the date of randomization.

The ORR is defined as the proportion of randomized subjects in each treatment arm whose best response is complete response (CR) or partial response (PR) according to the mRECIST for HCC. The DCR is defined as the proportion of randomized subjects in each treatment arm whose best response is CR, PR, or stable disease (SD) according to the modified RECIST for HCC (Table 1).

**Table 1:** Definitions of complete response (CR), partial response (PR), stable disease (SD) and progressive disease (PD)

Parameters	Definitions
Complete response	Complete disappearance of extrahepatic metastatic lesions under the contrast-agent enhancement in the arterial phase of spiral computed tomography (CT) or magnetic resonance imaging (MRI). CR must be confirmed by a second evaluation no less than 4 weeks after the date the CR was first obtained
Partial response	Decrease of > 30% in the sum of the longest diameters (SLD) of extrahepatic metastatic lesions with reference to the baseline SLD. PR must be confirmed by a second evaluation no less than 4 weeks after the date the PR was first obtained
Stable disease	Failure to meet the criteria for complete or partial response, in the absence of progressive disease
Progressive disease	Increase of > 20% in the SLD of extrahepatic metastatic lesions with reference to the smallest SLD of target lesions recorded since the treatment started. If the 20% or more increase is observed in two consecutive determinations, the date of PD is the date of the first evaluation; or New extrahepatic metastatic lesions with the longest diameter of at least 10 mm confirmed by contrast-enhanced spiral CT or MRI imaging. or Appearance of one or more new extrahepatic lesions of any size, or Appearance of more intrahepatic lesions, expanding existing lesions (including portal vein emboli)

The DR is defined as the time from randomization to disease progression or death in randomized subjects whose best response is CR or PR. Subjects who neither progress nor die will be censored on the date of their last tumor assessment. The TR is defined as the time from randomization to the time when the response criteria are met for CR or PR, whichever occurs first. Time to response is computed only for subjects whose best response is CR or PR.

QoL will be evaluated at every visit using the European Organization for Research and Treatment of Cancer (EORTC) QLQ-C30 version 3.0 questionnaire [19, 20]. The QLQ-C30 consists of 30 questions on a 5-point scale that ask subjects to assess the degree to which they were bothered by health-related quality of life (HRQoL) issues in the last 7 days at the time the question is answered. The QLQ-C30 consists of physical, role, social, and emotional functioning domains. The QLQ-C30 is validated for use in multiple types of cancer and is the most widely used HRQoL instrument in oncology clinical trials to date. This instrument has been used in studies of HRQoL in HCC; baseline scores for role and physical functioning domains were demonstrated to predict survival in subjects with advanced HCC [21]. The QLQ-C30 is currently being validated in a global population of patients with HCC [22]. The QLQ-C30 will be self-administered by subjects at baseline, at every 4-week clinic visit, and at the end-of-treatment visit. These self-assessments will be conducted by research coordinators prior to clinic staff interaction, to avoid bias.

### Safety endpoints

Study drug toxicity will be assessed continuously, including monitoring of vital signs, physical examination, neurological examination, electrocardiogram (EKG), echo, clinical laboratory tests (hemoglobin, hematocrit, red blood cell, total leukocyte count with differential, platelet count, AST, ALT, total bilirubin, direct bilirubin, alkaline phosphatase, lactate dehydrogenase (LDH), creatinine, blood urea nitrogen (BUN) or urea, blood glucose, total protein, albumin, sodium, potassium, chloride, total calcium, phosphorus, magnesium, ammonia, and alpha-fetoprotein (AFP)) and specialty examinations (chest x-ray, CT, MRI, bone scan, etc.). All adverse events will be evaluated according to the NCI Common Terminology Criteria for Adverse Events (CTCAE) (Version 3.0), on a continuous basis while the subject is in the study. Scheduled evaluations will occur every 4 weeks.

## Safety reporting

### Adverse events

An adverse event (AE) is defined as any new untoward medical occurrence or worsening of a pre-existing medical condition in a patient or clinical investigation subject who is administered an investigational product and that does not necessarily have a causal relationship with this treatment. An AE can therefore be any unfavorable and unintended sign (including an abnormal laboratory finding, for example), symptom, or disease temporally associated with the use of the investigational product, whether or not it is considered related to the investigational product.

### Serious adverse events

Serious adverse events (SAEs) are any of the following untoward medical events:

- Death
- Life-threatening (defined as an event in which the subject was at risk of death)
- Inpatient hospitalization or prolongation of existing hospitalization
- Persistent or significant disability/incapacity
- Congenital anomaly/birth defects

### Judgement of AEs/SAEs

All AEs and SAEs must be graded according to the National Cancer CTCAE version 3, dated 9 Aug. 2006. The following are the categories and definitions of a causal relationship with the study drug as determined by a physician:

- Related: there is a reasonable causal relationship between study drug administration and AEs and SAEs.
- Not related: there is not a reasonable causal relationship between study drug administration and the AE and SAEs. The expression “reasonable causal relationship” is meant to convey in general that there are facts or other arguments to suggest a positive causal relationship.

### Collection and reporting

AEs can be spontaneously reported or elicited during open-ended questioning, examination, or evaluation of a subject (to prevent reporting bias, subjects should not be questioned about the specific occurrence of one or more AEs).

If known, the diagnosis of the underlying illness or disorder should be recorded, rather than its individual symptoms. The following information should be captured for all AEs: onset, duration, intensity, seriousness, relationship to study drug, action taken, and treatment required. If treatment for the AE is administered, it should be recorded on the appropriate case report file (CRF) page. The investigator shall supply the Sponsor and Ethics Committee with any additional requested information, notably on reported deaths of subjects. Completion of supplemental CRFs may be requested for AEs and/or laboratory abnormalities that are reported/identified during the course of the study.

## Statistical considerations

### Sample size estimation

The sample size is calculated to compare OS between two arms. We agree that an additional 10% OS after treatment with ATRA + FOLFOX would be beneficial to patients and clinically meaningful. Qin et al. [14] reported a 1-year survival rate of 15% after treatment with FOLFOX4 only, so we assumed ATRA + FOLFOX would give an additional 10% OS benefit (which is 25%). Therefore, with overall two-sided alpha of 5.0%, 320 patients (160 per group) are required to provide 80% power to detect a hazard ratio of 1.13 in median survival OR between the two treatment arms. Furthermore, considering a 15% loss to follow up, we need a total of 368 patients (184 patients per group).

### Populations for analysis

The intent-to-treat (ITT) population will comprise any patients assigned to a treatment group by the randomization process, regardless of whether patients received any study treatment or received a different study treatment to the one to which they were randomized. This is the primary data set for the analysis of OS, and of secondary efficacy endpoints like TTP, symptom assessment, and baseline characteristics.

The per protocol (PP) population will comprise all randomized subjects except for subjects who (1) are wrongly diagnosed with cancer; (2) are not treated; or (3) are not treated with the study therapy as assigned by the randomization. This is the primary dataset for the analysis of OS.

The safety population will comprise all subjects who received at least one dose of study medication. This is the primary data set for analysis of dosing, safety, concomitant medications.

### Analysis of efficacy

OS will be compared between two treatment groups, using the log-rank test procedure at 5% significance level and the survival curves will be estimated using the Kaplan-Meier method. Additional analyses of survival will include the computation of hazard ratios (HRs) and the estimation of survival function. The survival hazard ratio of TARA + FOLFOX to placebo + FOLFOX and the associated two-sided 95.0% confidence interval will be computed using unadjusted and adjusted Cox proportional hazards modeling. Cox models will include treatment stratified by the aforementioned stratification factors, and an adjusted model, which will include a pre-defined list of covariates (described in the next paragraph) and the aforementioned stratification factors, all as covariates. The response rate (CR and PR) will be compared between the two treatments using the chi-square test or Fisher’s exact test. Repeated measures analysis of variance (ANOVA) (if applicable) will be used or the two-group *t* test/Wilcoxon signed rank test on the difference between the baseline score and the best score in each scale will be used to compare quality of life between the two treatments.

### Analysis of safety

We will conduct a safety analysis on the safety population. Worst toxicity grades per subject will be tabulated for selected AEs and laboratory measurements. All recorded AEs, SAEs, and AEs leading to study therapy discontinuation will be listed and tabulated. Vital signs and clinical laboratory test results will be listed and summarized by treatment arm. Any significant physical examination findings, ECG results, and clinical laboratory results will be listed.

## Schedule of events 

The schedule of events is shown in Table 2.

**Table 2:** Schedule of events

Visits	Enrollment and Randomization	M1	M2	M3	M4	M5	M6	M7	M8	M9	M10	M11	M12
Identification	X												
Screen by inclusion/exclusion criteria	X												
Informed consent form	X												
Initial assessment (history/physical/laboratory/imaging)	X												
Assessment – History/physical	X	X	X	X	X	X	X	X	X	X	X	X	X
CT/MRI	X	X	X	X	X	X	X	X	X	X	X	X	X
Laboratory tests	X	X	X	X	X	X	X	X	X	X	X	X	X
Quality of life questionnaires	X	X	X	X	X	X	X	X	X	X	X	X	X
Overall survival		X	X	X	X	X	X	X	X	X	X	X	X
Tumor progression Assessment		X	X	X	X	X	X	X	X	X	X	X	X
Concomitant medication	X	X	X	X	X	X	X	X	X	X	X	X	X
AEs/SAEs		X	X	X	X	X	X	X	X	X	X	X	X

*M* month, *CT* computed tomography, *MRI* magnetic resonance imaging, *AE* adverse event, *SAE* serious adverse event

## Discussion

Among patients with HCC, 85% have an advanced disease stage at diagnosis, and a large number of patients diagnosed with early-stage disease eventually experience recurrence [23]. Sorafenib, the only drug that is approved by the Food and Drug Administration (FDA) for treatment of patients with advanced cancer according to the Barcelona Clinic Liver Cancer (BCLC) staging classification, extends overall survival by approximately 3 months. The response rate is only 2–3% and the acquisition of CR is rarer [24]. In contrast to other solid cancers, chemotherapy has not previously been used routinely in HCC because of the toxicity and high incidence of chemo-resistance.

However, with the constant progress of chemotherapeutics, especially platinum drugs, recent trials have proved that chemotherapy may be useful in the treatment of advanced HCC. In one recent study [25], metronomic chemotherapy including 5-FU and cisplatin was associated with more favorable outcomes in terms of overall survival as compared to sorafenib in advanced HCC (158 days vs 117 days, *p* = 0.029). In the EACH study [14], FOLFOX improved median overall survival compared with doxorubicin (6.40 months vs 4.97 months, *p* = 0.07) with a similar rate of AEs, and has been established as an option in the treatment of advanced HCC.

Despite the advances in systemic chemotherapy, drug resistance is still an obstacle to further improvement. Increasingly, evidence has shown that TICs exhibit greater resistance to conventional chemotherapies than non-TICs. It is well-known that ATRA is one of the strongest and the most thoroughly studied differentiation inducers, which may induce differentiation of different kinds of tumor cells including stem cells. Our previous studies [16, 17] showed that ATRA could induce differentiation of HCC TICs via the TS2/AKT pathway and combined treatment with ATRA and cisplatin could improve the therapeutic effect, due to elimination of TICs via ATRA-induced differentiation, in vivo and in vitro. Therefore, our hypothesis holds that treatment with ATRA/FOLFOX in extrahepatic metastasis might be useful; so far, no prospective trial has been conducted in this setting.

### Trial status

Patient recruitment has not yet started.

## Additional file

Additional file 1: SPIRIT 2013 checklist: Recommended items to address in a clinical trial protocol and related documents. (DOC 122 kb)

## Abbreviations

5FU: 5-Fluorouracil
AE: Adverse event
ALT: Glutamic-pyruvic transaminase
AST: Glutamic oxaloacetic transaminase
ATRA: All-trans-retinoic acid
BCLC: Barcelona Clinic Liver Cancer
CAD: Coronary atherothrombotic disease
CHF: Congestive heart failure
CR: Complete response
CRF: Case report file
CT: Computed tomography
DCR: Disease control rate
DR: Duration of response
FOLFOX: Oxaliplatin + 5-fluorouracil/leucovorin
HBV: Hepatitis B virus
HCC: Hepatocellular carcinoma
HRs: Hazard ratios
ITT: Intent-to-treat
LDH: Lactate dehydrogenase
LV: Leucovorin
mRECIST: Modified Response Evaluation Criteria in Solid Tumors
mOS: Median overall survival
MRI: Magnetic resonance imaging
ORR: Objective response rate
OS: Overall survival
OXA: Oxaliplatin
PD: Progressive disease
PFS: Progression-free survival
PIAF: Cisplatin, interferon α-2b, fluorouracil, and doxorubicin
PP: Per protocol
PR: Partial response
QoL: Quality of life
RR: Response rate
SAE: Serious adverse event
SBSC: Standard best supportive care
SD: Stable disease
TACE: Transhepatic arterial chemotherapy and embolization
TIC: Tumor-initiating cell
TR: Time to response
TTP: Time to progression
UNL: Upper normal limit
